# Activation of Galectin-3 (LGALS3) Transcription by Injurious Stimuli in the Liver Is Commonly Mediated by BRG1

**DOI:** 10.3389/fcell.2019.00310

**Published:** 2019-11-26

**Authors:** Zilong Li, Fangqiao Lv, Congxin Dai, Qiong Wang, Chao Jiang, Mingming Fang, Yong Xu

**Affiliations:** ^1^Department of Surgical Oncology, the Affiliated Hospital of Nanjing University of Chinese Medicine, Jiangsu Province Hospital of Traditional Chinese Medicine, Nanjing, China; ^2^Department of Clinical Medicine, Laboratory Center for Basic Medical Sciences, Jiangsu Health Vocational College, Nanjing, China; ^3^Key Laboratory of Targeted Intervention of Cardiovascular Disease and Collaborative Innovation Center for Cardiovascular Translational Medicine, Department of Pathophysiology, Nanjing Medical University, Nanjing, China; ^4^Department of Cell Biology, School of Basic Medical Sciences, Capital Medical University, Beijing, China; ^5^Department of Neurosurgery, Peking Union Medical College Hospital, Beijing, China; ^6^Institute of Biomedical Research, Liaocheng University, Liaocheng, China

**Keywords:** transcriptional regulation, epigenetics, liver injury, hepatocytes, DNA demethylation

## Abstract

Galectin-3 (encoded by *LGALS3*) is a glycan-binding protein that regulates a diverse range of pathophysiological processes contributing to the pathogenesis of human diseases. Previous studies have found that galectin-3 levels are up-regulated in the liver by a host of different injurious stimuli. The underlying epigenetic mechanism, however, is unclear. Here we report that conditional knockout of Brahma related gene (BRG1), a chromatin remodeling protein, in hepatocytes attenuated induction of galectin-3 expression in several different animal models of liver injury. Similarly, BRG1 depletion or pharmaceutical inhibition in cultured hepatocytes suppressed the induction of galectin-3 expression by treatment with LPS plus free fatty acid (palmitate). Further analysis revealed that BRG1 interacted with AP-1 to bind to the proximal galectin-3 promoter and activate transcription. Mechanistically, DNA demethylation surrounding the galectin-3 promoter appeared to be a rate-limiting step in BRG1-mediated activation of galectin-3 transcription. BRG1 recruited the DNA 5-methylcytosine dioxygenase TET1 to the galectin-3 to promote active DNA demethylation thereby activating galectin-3 transcription. Finally, TET1 silencing abrogated induction of galectin-3 expression by LPS plus palmitate in cultured hepatocytes. In conclusion, our data unveil a novel epigenetic pathway that contributes to injury-associated activation of galectin-3 transcription in hepatocytes.

## Introduction

Transcriptional regulation in mammalian cells is acutely influenced by the epigenetic machinery, which includes DNA and histone modifying enzymes, chromatin remodeling proteins, and non-coding regulatory RNAs (Ledford, 2015). DNA is wrapped around histone octamers (nucleosomes) to form higher-order chromatins serving as basic transcription unit. A host of chemical groups are superimposed on DNA and histone tails (thus the prefix “epi-”) creating a layer of regulatory information (Shafabakhsh et al., 2019). Creation, maintenance, and recognition of this regulatory layer are coordinately mediated by the aforementioned epigenetic factors to dictate specific transcriptional events and program pathophysiological processes. Typically, chromatins can be categorized into three groups based on characteristic DNA/histone modifications associated with the promoter region (Lennartsson and Ekwall, 2009). Actively transcribed chromatins are marked by high levels of histone H3 acetylation and H3K4 trimethylation. On the contrary, silenced chromatin regions are demarcated by high levels of DNA methylation (CpG) and histone H3K9/H3K27 methylation. The third group of chromatins possess both the “active” and the “repressive” DNA/histone markers and therefore can be said to be labeled by “bivalent” modifications; removal of the “repressive” modifications potentiates transcription whereas erasure of the “active” modifications permanently turns off transcription (Harikumar and Meshorer, 2015).

Brahma related gene (BRG1) is the core component of the mammalian SWI/SNF chromatin remodeling complex (Wu H. et al., 2017). Along with its closely related sibling Brahma (BRM), BRG1 provides the ATPase activity to furrow the nucleosomes (Reisman et al., 2009). BRG1 contains a bromodomain that recognizes acetylated histones and helps tether BRG1 to the chromatin although locus-specific recruitment of BRG1 is determined by sequence-specific transcription factors (Ptashne, 2007, 2013). In addition to its role as a fueling force for nucleosomal mobilization, BRG1 can regulate transcription independent of its ATPase activity (Chaiyachati et al., 2013). Mounting evidence suggests that there are extensive dialogues between BRG1 and other branches of the epigenetic machinery. For instance, long non-coding RNAs (lncRNAs) can interact with BRG1 and modulate BRG1 activity (Leisegang et al., 2017; Chen et al., 2018; Huang et al., 2019). BRG1 has also been shown to form crosstalk with histone acetyltransferases/deacetylases, histone methyltransferases/demethylases, and DNA methyltransferases/demethylases (Pal et al., 2004; Naidu et al., 2009; Yildirim et al., 2011; Xiao et al., 2016; Zhang et al., 2018; Shao et al., 2019). Owing to its pivotal role in transcriptional regulation, BRG1 deficiency causes developmental arrest and premature death during embryogenesis (Bultman et al., 2000, 2005; Griffin et al., 2008, 2011). There is emerging evidence that aberrant activation of BRG1 may result in the pathogenesis of a host of human diseases including atherosclerosis (Fang et al., 2013), pulmonary arterial hypertension (Chen et al., 2013), non-alcoholic steatohepatitis (Tian et al., 2013), cardiac hypertrophy (Hang et al., 2010), and cancers (Wu Q. et al., 2017).

Galectin-3, encoded by *LGALS3*, belongs to a family of glycan-binding proteins regulating such fundamental cellular functions as proliferation, migration, differentiation, apoptosis, and inflammation (Sciacchitano et al., 2018). Galectin-3 exhibits a ubiquitous expression pattern in adults under physiological conditions. When exposed to injurious stimuli, expression of galectin-3 can be altered in a cell type and signal-specific manner indicating it may serve as an “alarmin” that mediates cellular stress responses. For instance, it has previously been reported that hepatic galectin-3 expression is up-regulated in different animal models of liver injury but the underlying epigenetic mechanism remains underappreciated (Henderson et al., 2006; Dragomir et al., 2012b; Pejnovic et al., 2013; Locatelli et al., 2014; Jeftic et al., 2015). Here we report that activation of galectin-3 transcription by different injurious stimuli in the liver shares a common mediator in BRG1. BRG1 directly binds to the galectin-3 promoter by interacting with AP-1 and recruits the DNA demethylase (dioxygenase) TET1 to activate galectin-3 transcription.

## Materials and Methods

### Animals

All animal experiments were reviewed and approved by the intramural Ethics Committee on Humane Treatment of Experimental Animals. The breedings were conducted by Nanjing Biomedical Research Institute of Nanjing University. Hepatocyte conditional BRG1 knockout (CKO) mice were obtained by crossing the *Smarca4*^f/f^ mice (Eroglu et al., 2006) with the *Alb*-Cre mice (Jackson Laboratory). To induce liver injury, 6∼8 week male mice were fasted overnight before receiving a single injection of APAP (Sigma) at 300 mg/kg. Alternatively, 6∼8 week male mice were given a single injection of CCl_4_ (1.0 mL/kg body weight as 50%, vol/vol) as previously described (Li et al., 2018a). To induce steatotic injury, 8 week-old male were fed a methionine- and choline-deficient (MCD) diet (A02082002B, Research Diets) for eight consecutive weeks as previously described (Liu et al., 2019a). Alternatively, the mice were fed on a high-fat diet (HFD, D12492, Research Diets) for 16 weeks as previously described (Li et al., 2018b). To silence BRG1 in *db*/*db* mice, the mice were injected via tail vein purified lentiviral particles (1 × 10^9^ MOI) that carry short hairpin RNA (shRNA) targeting Brg1 (GCUGGAGAAGCAGCAGAAG) as previously described (Tian et al., 2013).

### Cell Culture, Plasmids, Transient Transfection, and Reporter Assay

Primary mouse hepatocytes were isolated and maintained as previously described (Fan et al., 2017). HepG2 cells were maintained in DMEM supplemented with 10% FBS. FLAG-tagged BRG1 (Li et al., 2018e) and LGALS3 promoter-luciferase constructs (Kadrofske et al., 1998) have been described previously. LPS (1 μg/ml) and palmitate (0.4 mM) were purchased from Sigma. Small interfering RNAs were purchased from Dharmacon. Transient transfection was performed with Lipofectamine 2000. Briefly, cells were plated in 12-well culture dishes (∼60,000 cells/well). The next day, 0.1 μg of reporter construct and 0.1–0.3 μg of effector construct were transfected into each well. DNA content was normalized by the addition of an empty vector (pcDNA3). For monitoring transfection efficiency and for normalizing luciferase activity, 0.02 μg of GFP construct was transfected into each well. Cells were harvested 48 hours after transfection and reporter activity was measured using a luciferase reporter assay system (Promega) as previously described (Liu et al., 2018). All experiments were repeated three times.

### RNA Isolation and Real-Time PCR

RNA was extracted with the RNeasy RNA isolation kit (Qiagen). Reverse transcriptase reactions were performed as previously described using a SuperScript First-strand Synthesis System (Invitrogen) (Zeng et al., 2018). Real-time qPCR reactions were performed in triplicate wells on an ABI StepOnePlus (Life Tech). The relative quantification for a given gene was normalized by the *Gapdh* mRNA values. All experiments were repeated three times.

### Protein Extraction, Immunoprecipitation, and Western Blotting

Whole cell lysates were obtained by re-suspending cell pellets in RIPA buffer with freshly added protease inhibitor tablet (Roche). Immunoprecipitation was performed essentially as previously described (Yang et al., 2018). Briefly, anti-Brg1 (Santa Cruz, sc-17796), or pre-immune IgGs (P.I.I.) were added to and incubated with cell lysates overnight before being absorbed by Protein A/G-plus Agarose beads (Santa Cruz). Precipitated immune complex was released by boiling with 1X SDS electrophoresis sample buffer. Western analyses were performed with anti-β-actin (Sigma), anti-Brg1 (Santa Cruz, sc-17796), anti-TET1 (Active Motif, 61443), anti-c-Jun (Santa Cruz, sc-1694), anti-c-Fos (Santa Cruz, sc-52), or anti-Galectin-3 (Proteintech, 14979-1). Uncropped full blots are included in the Supplementary Material (Supplementary Figures S1–S4).

### Chromatin Immunoprecipitation (ChIP)

Chromatin immunoprecipitation assays were performed essentially as described before (Li et al., 2017, 2018c,d, 2019b; Yu et al., 2017, 2018; Fan et al., 2019; Kong et al., 2019a, b; Liu et al., 2019b; Weng et al., 2019; Yang et al., 2019a, b; Zhang et al., 2019). In brief, chromatin in control and treated cells were cross-linked with 1% formaldehyde. Cells were incubated in lysis buffer (150 mM NaCl, 25 mM Tris pH 7.5, 1% Triton X-100, 0.1% SDS, 0.5% deoxycholate) supplemented with protease inhibitor tablet and PMSF. DNA was fragmented into ∼500 bp pieces using a Branson 250 sonicator. Aliquots of lysates containing 200 μg of protein were used for each immunoprecipitation reaction with the following antibodies: anti-Brg1 (Santa Cruz, sc-17796), anti-acetyl histone H3 (Millipore, 06-599), anti-trimethyl H3K4 (Millipore, 07-473), anti-dimethyl H3K9 (Millipore, 07-441), anti-5′-hydroxymethylcytosine (Abcam, ab106918), anti-5′-methylcytosine (Abcam, ab10805), anti-TET1 (Active Motif, 61443), anti-TET2 (Millipore, MABE462), anti-TET3 (ABE290), anti-c-Jun (Santa Cruz, sc-1694), anti-c-Fos (Santa Cruz, sc-52), or IgG. Precipitated DNA was amplified with the following primers: for *LGALS3* promoter (-402/-73), 5′-AATTTGTAGTCAGTTCCCTAG-3′ and 5′-AAATACTCCCAGCCCCGC-3; for *LGALS3* promoter (-1276/-959), 5′-ATACCTGGTTTTCTCCATAG-3′ and 5′-ATATTGCCTATAAGCTACCC-3′.

### Statistical Analysis

Data are presented as mean ± SEM. For experiments concerning multiple groups, one-way ANOVA with *post hoc* Scheffe analyses were performed to evaluate the differences using an SPSS package (IBM analytics). The differences between two (control and experimental) groups were determined by two-sided, unpaired Student’s *t*-test.

## Results

### BRG1 Regulates Injury Induced Galectin-3 Up-Regulation in the Liver

**FIGURE 1:**
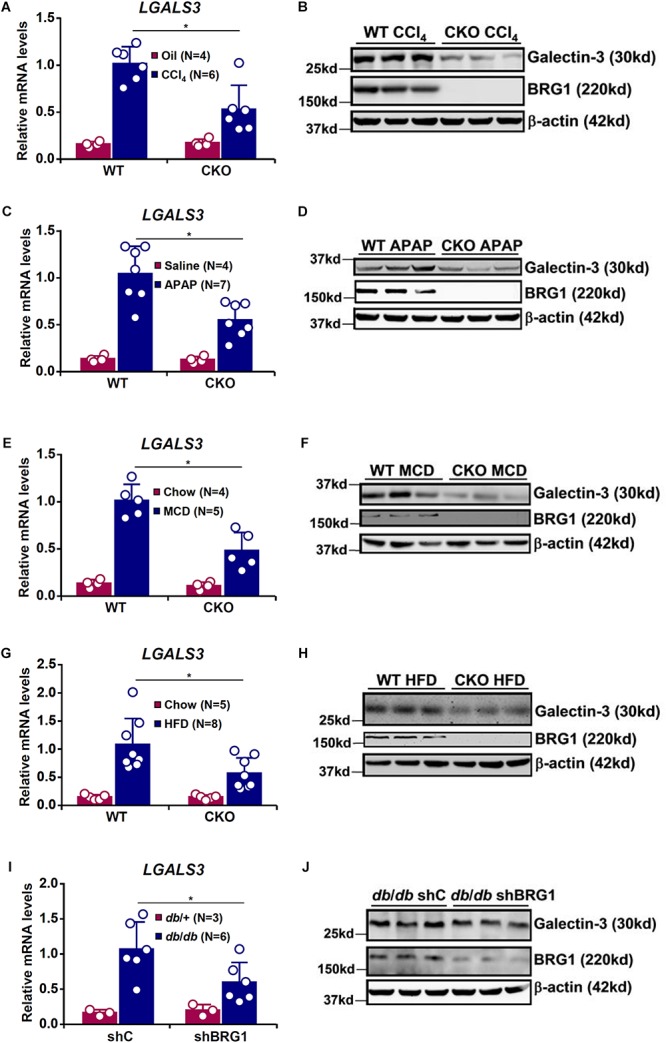
BRG1 regulates injury induced galectin-3 up-regulation in the liver. **(A,B)** WT and CKO mice were given weekly injection of corn oil or CCl_4_ for 6 weeks. Hepatic expression of galectin-3 was examined by qPCR and Western. **(C,D)** WT and CKO mice were injected with saline or APAP (300 mg/kg) and sacrificed 48 h after the injection. Hepatic expression of galectin-3 was examined by qPCR and Western. **(E,F)** WT and CKO mice were fed an MCD diet or a control diet for 8 weeks. Hepatic expression of galectin-3 was examined by qPCR and Western. **(G,H)** WT and CKO mice were fed an HFD diet or a control diet for 8 weeks. Hepatic expression of galectin-3 was examined by qPCR and Western. **(I,J)** *Db*/*db* mice and *db*/ + mice were injected with lentivirus carrying shRNA targeting BRG1 or a control shRNA. Hepatic expression of galectin-3 was examined by qPCR and Western. Error bars represent SD (^∗^*p* < 0.05, one-way ANOVA with *post hoc* Scheffe test).

It has previously been demonstrated that hepatic galectin-3 expression is up-regulated in different animal models of liver injury (Henderson et al., 2006; Dragomir et al., 2012b; Pejnovic et al., 2013; Locatelli et al., 2014; Jeftic et al., 2015). On the other hand, we have shown that BRG1 deficiency attenuates liver injuries induced by toxin (Li et al., 2019a), drug overdose (Li et al., 2018a), or diet (Kong et al., 2018; Li et al., 2018b; Liu et al., 2019a) in mice. We hypothesized that BRG1 might play a role in regulating galectin-3 expression. To test this hypothesis, hepatocyte conditional BRG1 knockout (CKO) mice and wild type (WT) mice were subjected to different treatments of liver injury. In the first model, weekly injection of CCl_4_ was administered for 6 weeks. Hepatic galectin-3 expression was significantly up-regulated in CCl_4_-injected mice at the mRNA (Figure 1A) and protein (Figure 1B) levels; galectin-3 induction was less robust in CKO mice than in WT mice. In the second model, the mice were injected with a single dose of acetaminophen (APAP) to induce acute liver injury. APAP injection resulted in significant up-regulation of galectin-3 expression in both WT and CKO mice although CKO mice displayed more modest up-regulation of galectin-3 (Figures 1C,D).

Next, we exploited two different models of diet-induced steatotic injury. BRG1 deficiency compromised galectin-3 induction by MCD diet feeding (Figures 1E,F) or HFD diet feeding (Figures 1G,H). Lastly, we examined the effect of BRG1 deficiency on galectin-3 expression in a genetically predisposed model of steatotic injury. ShRNA mediated knockdown of BRG1 in *db*/*db* mice suppressed galectin-3 expression compared to the control *db*/*db* mice (Figures 1I,J). Thus, it appears that induction of galectin-3 by injurious stimuli in the liver may share a common mediator.

### BRG1 Regulates Galectin-3 Up-Regulation in Cultured Hepatocytes

**FIGURE 2:**
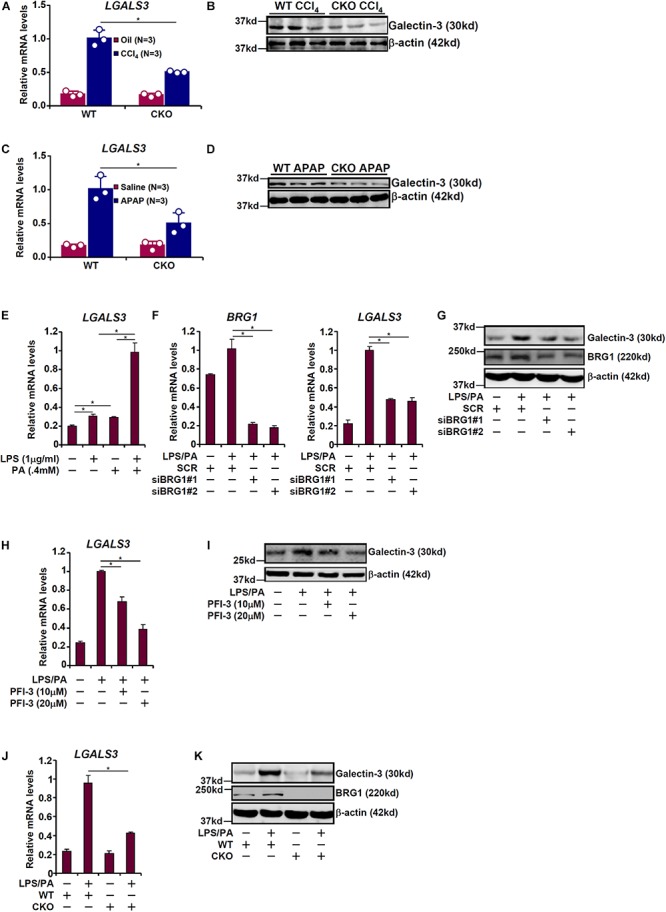
BRG1 regulates galectin-3 up-regulation in cultured hepatocytes. **(A,B)** WT and CKO mice were given weekly injection of corn oil or CCl_4_ for 6 weeks. Primary hepatocytes were isolated and expression of galectin-3 was examined by qPCR and Western. **(C,D)** WT and CKO mice were injected with saline or APAP (300 mg/kg) and sacrificed 48 h after the injection. Primary hepatocytes were isolated and expression of galectin-3 was examined by qPCR and Western. **(E)** HepG2 cells were treated with LPS and/or palmitate for 24 hours. Galectin-3 expression was examined by qPCR. **(F,G)** HepG2 cells were transfected with small interfering RNA targeting BRG1 or scrambled siRNA (SCR) followed by treatment with LPS plus palmitate. Galectin-3 expression was examined by qPCR and Western. **(H,I)** HepG2 cells were treated with LPS plus palmitate in the presence or absence of PFI-3. Galectin-3 expression was examined by qPCR and Western. **(J,K)** Primary hepatocytes were isolated from WT and CKO mice and treated with or without LPS plus palmitate. Galectin-3 expression was examined by qPCR and Western. Data represent averages of three independent experiments. Data represent averages of three independent experiments and error bars represent SEM (^∗^*p* < 0.05, one-way ANOVA with *post hoc* Scheffe test).

Galectin-3 has been shown to be primarily expressed in macrophages and the epithelium under physiological conditions but can be up-regulated in a wide range of cell types under pathological conditions (Chiariotti et al., 2002). To further verify whether galectin-3 expression may be up-regulated in hepatocytes exposed to injurious cues, primary hepatocytes were isolated from the mice before and after the exposure to various injurious stimuli. BRG1 deficiency attenuated galectin-3 induction by either CCl_4_ injection (Figures 2A,B) or APAP injection (Figures 2C,D) in hepatocytes.

The various mouse models of liver injuries as described above differ significantly in etiology and pathogenesis. Inflammatory response as a result of activation of the innate immune system and increased serum free fatty acid levels due to a combination of augmented lipolysis and impaired β-oxidation can be said to be common to all these animal models (Stern et al., 1965; Begriche et al., 2011; Sandhir et al., 2017; Strazzabosco et al., 2018; Visentin et al., 2018). Therefore, we sought to determine whether BRG1 deficiency in hepatocytes would similarly regulate galectin-3 expression in response to LPS and free fatty acids. As shown in Figure 2E, treatment with LPS (1 μg/ml) or palmitate (0.4 mM) led to a small but significant up-regulation of galectin-3 expression in HepG2 cells; combined treatment of LPS and palmitate, however, evoked a much stronger induction of galectin-3 expression. Therefore, LPS plus palmitate was used to stimulate galectin-3 expression in cultured cells hereafter. Depletion of BRG1 with siRNAs markedly suppressed induction of galectin-3 expression by LPS plus palmitate (Figures 2F,G). Likewise, inhibition of BRG1 activity with a small-molecule compound (PFI-3) dose-dependently reversed induction of galectin-3 expression by LPS plus palmitate (Figures 2H,I). Finally, when primary hepatocytes were isolated from both WT and CKO mice and treated with LPS plus palmitate, the induction of galectin-3 expression was much stronger in WT cells than in CKO cells (Figures 2J,K). Of note, galectin-3 expression was still inducible, even though not quite as strong as WT cells, in BRG1 CKO cells by LPS plus palmitate indicative of a BRG1-independent mechanism.

### BRG1 Directly Binds to the Galectin-3 Promoter to Activate Transcription

**FIGURE 3:**
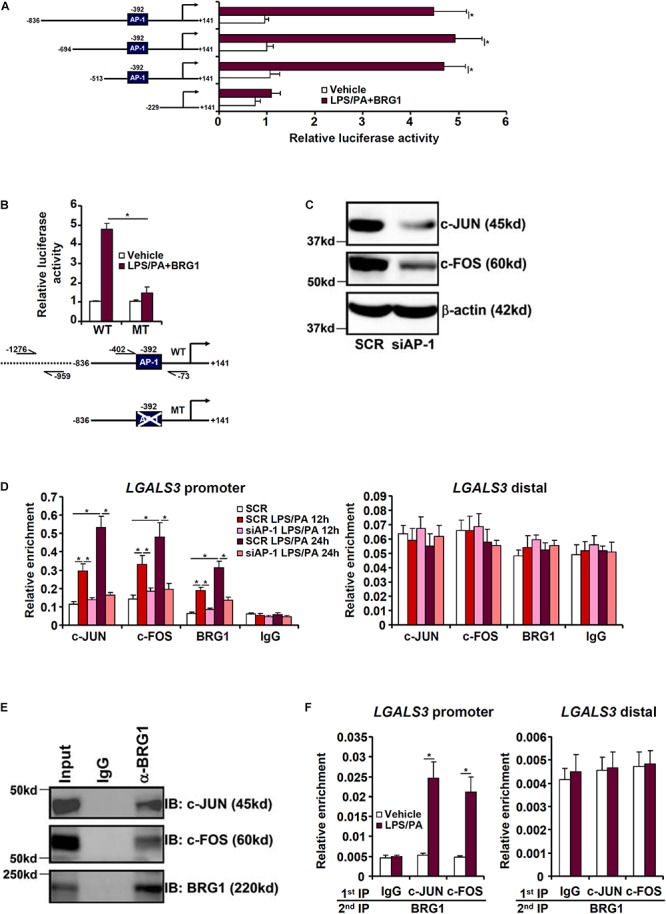
BRG1 interacts with AP-1 to activate galectin-3 transcription in hepatocytes. **(A)** Galectin-3 promoter-luciferase constructs of varying lengths were transfected into HepG2 cells with or without BRG1 followed by treatment with LPS plus palmitate. Luciferase activities were normalized to both GFP fluorescence and protein concentration. **(B)** Wild type or AP-1 site mutated galectin-3 promoter-luciferase construct was transfected into HepG2 cells with or without BRG1 followed by treatment with LPS plus palmitate. Luciferase activities were normalized to both GFP fluorescence and protein concentration. **(C,D)** HepG2 cells were transfected with siRNA targeting AP-1 or SCR followed by treatment with LPS plus palmitate. ChIP assays were performed with indicated antibodies. **(E)** HepG2 cells were treated with LPS plus palmitate. Nuclear proteins were extracted and immunoprecipitated with indicated antibodies. **(F)** HepG2 cells were treated with or without LPS plus palmitate. Re-ChIP assays were performed with indicated antibodies. Data represent averages of three independent experiments and error bars represent SEM (^∗^*p* < 0.05, one-way ANOVA with *post hoc* Scheffe test).

We next investigated how BRG1 could be recruited to the galectin-3 promoter in response to injurious stimuli. A series of galectin-3 promoter-luciferase constructs with progressively inward deletions were transfected into HepG2 cells. Over-expression of BRG1 in the presence of LPS plus palmitate markedly augmented the galectin-3 promoter activity until the deletion extended beyond a region containing an AP-1 binding site (Figure 3A). Indeed, mutation of this AP-1 site abrogated the induction of galectin-3 promoter activity by BRG1 over-expression (Figure 3B). ChIP assays showed that when the cells were exposed to the injurious stimuli, both AP-1 and BRG1 were recruited to the same region of the galectin-3 promoter with comparable kinetics; depletion of AP-1 with siRNAs significantly diminished BRG1 binding to the galectin-3 promoter (Figures 3C,D). Co-immunoprecipitation assay confirmed that BRG1 interacted with AP-1 in HepG2 cells (Figure 3E). More important, Re-ChIP assay detected the presence of a BRG1-AP-1 complex on the galectin-3 promoter only when the cells were stimulated with LPS plus palmitate (Figure 3F). Together, these data suggest that BRG1 may rely on AP-1 to activate galectin-3 transcription.

### BRG1 Regulates DNA Demethylation on the Galectin-3 Promoter

**FIGURE 4:**
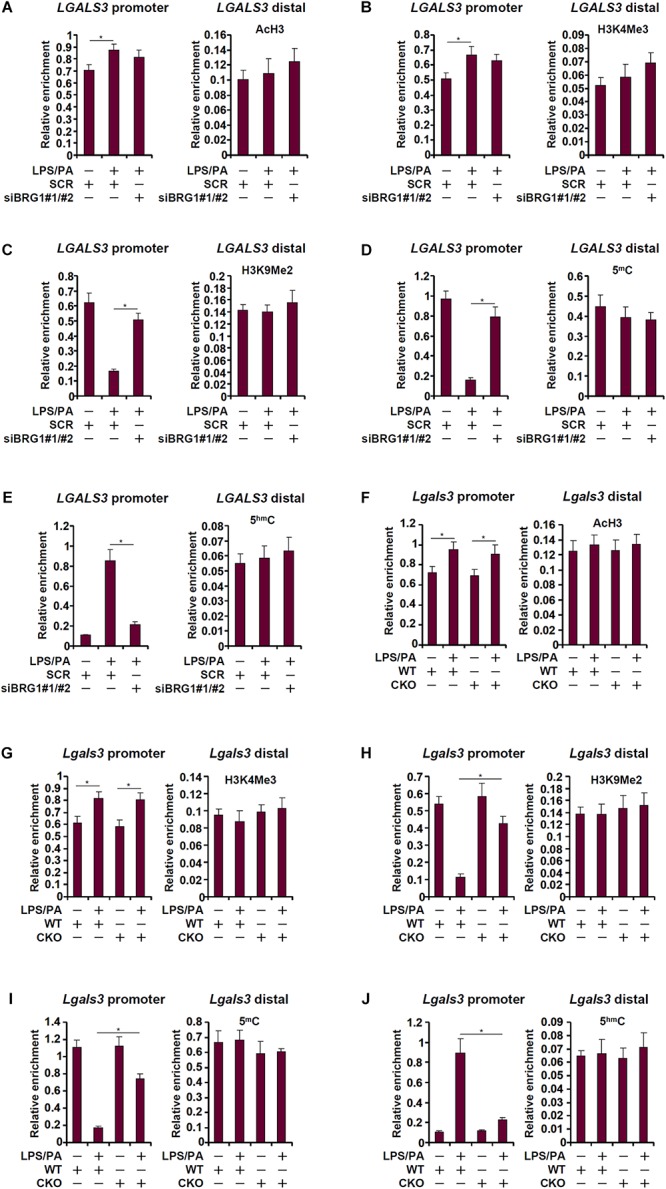
BRG1 regulates DNA demethylation on the galectin-3 promoter. **(A–E)** HepG2 cells were transfected with siRNA targeting BRG1 or SCR followed by treatment with LPS plus palmitate. ChIP assays were performed with anti-acetyl H3 **(A)**, anti-trimethyl H3K4 **(B)**, anti-trimethyl H3K9 **(C)**, anti-5′-methylcytosine **(D)**, and anti-5′-hydroxylcytosine **(E)**. **(F–J)** Primary hepatocytes were isolated from WT and CKO mice and treated with or without LPS plus palmitate. ChIP assays were performed with anti-acetyl H3 **(F)**, anti-trimethyl H3K4 **(G)**, anti-trimethyl H3K27 **(H)**, anti-5′-methylcytosine **(I)**, and anti-5′-hydroxylcytosine **(J)**. Data represent averages of three independent experiments and error bars represent SEM (^∗^*p* < 0.05, one-way ANOVA with *post hoc* Scheffe test).

We next determined the epigenetic mechanism whereby BRG1 may contribute to galectin-3 transcription. ChIP assays revealed that in response to treatment with LPS plus palmitate, levels of acetylated histone H3 (AcH3, Figure 4A) and trimethylated H3K4 (H3K4Me3, Figure 4B), both of which are well-established markers for active transcription, were slightly up-regulated on the galectin-3 promoter; BRG1 knockdown marginally influenced AcH3 and H3K4Me3 levels. On the contrary, levels of dimethylated H3K9 (H3K9Me2, Figure 4C), known to mark repressed chromatin, were significantly down-regulated on the galectin-3 promoter upon the addition of LPS plus palmitate; BRG1 depletion abolished the erasure of H3K9Me2 from the galectin-3 promoter. Further, there was a simultaneous disappearance of 5-methylcytosine (Figure 4D) and acquisition of 5-hydroxymethylcytosine (Figure 4E) on the galectin-3 promoter paralleling its trans-activation, a trend that was reversed by the loss of BRG1.

**FIGURE 5:**
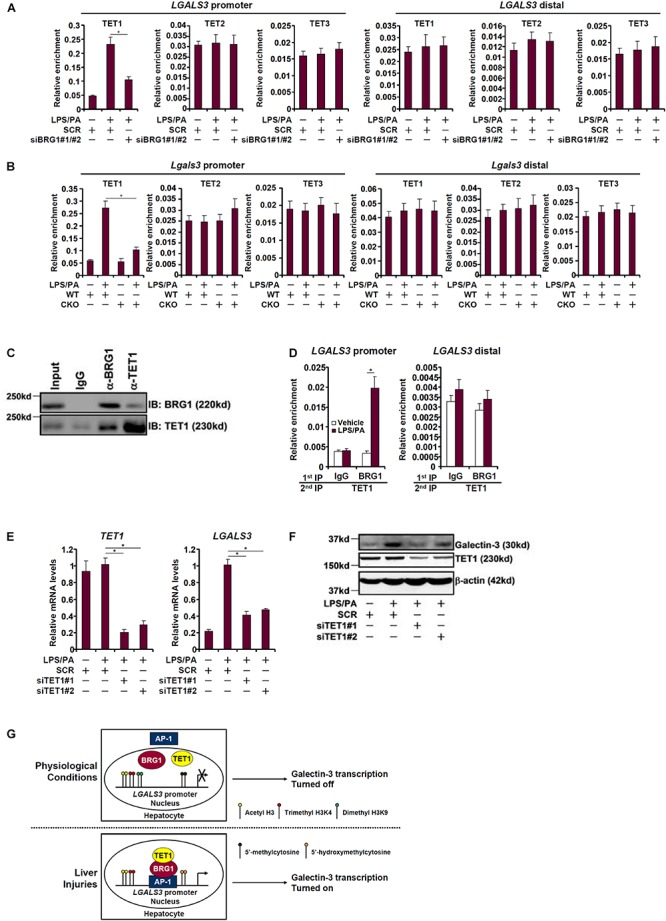
BRG1 cooperates with TET1 to activate galectin-3 transcription. **(A)** HepG2 cells were transfected with siRNA targeting BRG1 or SCR followed by treatment with LPS plus palmitate. ChIP assays were performed with indicated antibodies. **(B)** Primary hepatocytes were isolated from WT and CKO mice and treated with or without LPS plus palmitate. ChIP assays were performed with indicated antibodies. **(C)** HepG2 cells were treated with LPS plus palmitate. Nuclear proteins were extracted and immunoprecipitated with indicated antibodies. **(D)** HepG2 cells were treated with or without LPS plus palmitate. Re-ChIP assays were performed with indicated antibodies. **(E,F)** HepG2 cells were transfected with siRNA targeting TET1 or SCR followed by treatment with LPS plus palmitate. Galectin-3 expression was examined by qPCR and Western. Data represent averages of three independent experiments. Data represent averages of three independent experiments and error bars represent SEM (^∗^*p* < 0.05, one-way ANOVA with *post hoc* Scheffe test). **(G)** A schematic model.

We performed similar experiments using primary hepatocytes isolated from WT and CKO mice. AcH3 (Figure 4F) and H3K4Me3 (Figure 4G) levels were minimally altered by the treatment of LPS plus palmitate with or without BRG1. In sharp contrast, BRG1 deficiency was associated with the re-appearance of H3K9Me2 (Figure 4H) and 5-methylcytosine (Figure 4I) as well as the suppression of 5-hydroxymethylcytosine (Figure 4J). Combined, these data suggest that BRG1-dependent removal of repressive epigenetic traits on the galectin-3 promoter may be the rate-limiting step for its trans-activation.

### BRG1 Cooperates With TET1 to Activate Galectin-3 Transcription

Because BRG1 appeared to be essential for DNA (cytosine) demethylation on the galectin-3 promoter we naturally hypothesized that it may interact and recruit the TET family of dioxygenases to the galectin-3 promoter. As shown in Figure 5A, TET1, but not TET2 or TET3, occupied the galectin-3 promoter with significant affinity when HepG2 cells were treated with LPS plus palmitate; BRG1 knockdown dampened TET1 binding without altering the binding patterns of either TET2 or TET3. Similarly, LPS plus palmitate treatment promoted TET1 recruitment to the galectin-3 promoter in primary hepatocytes isolated from WT mice but not from CKO mice (Figure 5B), confirming the essential role of BRG1 in recruiting TET1. Co-immunoprecipitation experiments provided additional evidence that there was an interaction between BRG1 and TET1 in hepatocytes (Figure 5C). Re-ChIP assays confirmed that a BRG1-TET1 complex was clearly detectable on the galectin-3 promoter following the stimulation with LPS plus palmitate (Figure 5D). Finally, we asked whether TET1 might be essential for galectin-3 induction. Knockdown of TET1 with two different siRNAs comparably suppressed the induction of both mRNA (Figure 5E) and protein (Figure 5F) levels of galectin-3 by LPS plus palmitate. We therefore conclude that injurious stimuli induced galectin-3 expression in hepatocytes may be attributable to an interplay between BRG1 and TET1.

## Discussion

Galectin-3 levels are sensitive to the disruption of cellular homeostasis (Mishra et al., 2013; Venkatraman et al., 2018). Here we detail a novel epigenetic pathway wherein the chromatin remodeling protein BRG1 contributes to galectin-3 induction by injurious stimuli in hepatocytes by the DNA demethylase (dioxygenase) TET1 (Figure 5G). Of interest, fairly high levels of acetyl histone H3 and trimethyl H3K4 were present on the galectin-3 promoter under basal conditions, which were slightly augmented upon stimulation with injurious treatment and marginally decreased by BRG1 silencing (Figure 4). Instead, BRG1 regulated galectin-3 transcription primarily by facilitating the removal of repressive modifications including dimethyl H3K9 and 5′-methylcytosine from the galectin-3 promoter. This observation contrasts previous findings where BRG1 has been shown to regulate transcription by helping recruit histone acetyltransferases and H3K4 methyltransferases (Fang et al., 2013; Li et al., 2018a, b; Zhang et al., 2018). On the other hand, Sepulveda et al. (2017a, b) have reported that BRG1 may regulate osteoblast differentiation by contributing to the activation of genes with bivalent promoters. In fact, genome-wide profiling of BRG1 target genes in eight cell lines derived from malignant rhabdoid tumors demonstrates that fewer than 10% are bivalent (Nakayama et al., 2017). These data combined allude to the versatility of BRG1 in terms of promoter selection.

There are several lingering issues that need to be addressed in future investigations. First, although our data indicate that activation of galectin-3 transcription was also accompanied by erasure of dimethyl H3K9, in addition of DNA demethylation, in a BRG1-dependent manner, the identity of the responsible H3K9 demethylase remains to be investigated. We have previously shown that BRG1 interacts with the H3K9 demethylase KDM3A to activate colony stimulating factor (CSF1) transcription in endothelial cells (Zhang et al., 2018; Shao et al., 2019). KDM3A has been proposed as a stress response factor in a number of diseased states. For instance, KDM3A can sense low oxygen tension (hypoxia) in hepatocytes to regulate cell proliferation, migration, and invasion (Beyer et al., 2008; Yamada et al., 2012; Park et al., 2013). It would be of great interest to determine whether KDM3A may contribute to galectin-3 transcription in hepatocytes and, by extension, liver injury. Second, how BRG1 itself is regulated by divergent injurious stimuli to activate transcription was not examined in the present study. Several different factors may collectively contribute to signal-dependent activation of BRG1. BRG1 is predominantly nucleus-bound so its recruitment to target promoters largely depends on the availability of sequence-specific transcription factors. We show here that AP-1, the nuclear translocation of which can be stimulated by LPS and palmitate (Guha et al., 2001; Cazanave et al., 2010), interacted with BRG1 and recruited BRG1 to the galectin-3 promoter. It was also noted that expression levels of BRG1 was slightly but significantly up-regulated by injurious stimuli (Figures 2C,G) that may account for the overall augmented BRG1 activity although it is unclear whether this change was solely the result of increased transcription rate or enhanced protein stability or a combination of both. The human *SMARCA4* promoter encoding BRG1 has yet to be functionally characterized. Coira et al. (2015) have recently reported that a group of miRNAs can target the 3′ untranslated region of the *SMARCA4* mRNA to down-regulate BRG1 expression in human lung cancer cells. In addition, specific ubiquitin ligases have been also been identified for BRG1 (Sohn et al., 2007; Huang et al., 2018). Clearly further investigations are warranted to clarify whether these mechanisms may contribute to signal-induced activation of BRG1. Finally, although we focused our investigation on the regulation of galectin-3 transcription in hepatocytes it should be pointed out that galectin-3 expression has been detected in other liver cell types. For instance, Henderson et al. have observed that galectin-3 expression is significantly up-regulated in hepatic stellate cells (HSCs) as they transition from a quiescent phenotype to an activated phenotype (Henderson et al., 2006). Dragomir et al. have reported that galectin-3 expression in macrophages can be induced by APAP in mice, which is associated with a pro-inflammatory phenotype switch of macrophages (Dragomir et al., 2012a). More recently, it has been suggested that galectin-3 expression in down-regulated in CD68 + macrophages but up-regulated in a-SMA^+^ cells in the liver in children diagnosed with steatosis (de Oliveira et al., 2019). Therefore, although hepatocytes constitute the bulk of the liver mass, other cell populations, including Kupffer cells, cuboidal epithelial cells, fibroblasts, sinusoidal endothelial cells, intrahepatic lymphocytes, and HSCs, may produce galectin-3 molecules that contribute to the overall pool of galectin-3 in the liver. It remains to be tested whether the model as proposed here may be applied to other cell types in the liver exposed to injurious stimuli.

We provide evidence to implicate an interaction between BRG1 and the DNA dioxygenase TET1 in the induction of galectin-3 expression. Fazzio and colleagues have previously shown that BRG1 and TET1 can be purified from the same mega-protein complex in embryonic stem cells (ESCs) (Yildirim et al., 2011). Coincidentally, BRG1 and TET1 are shown to co-regulate a subset of bivalent promoters in ESCs. Of interest, TET1 depletion appears to influence the recruitment of BRG1 to target promoters suggestive of an inter-dependence between BRG1 and TET1 in regulating gene transcription (Yildirim et al., 2011). Curiously, both TET2 (Sepulveda et al., 2017b) and TET3 (Lan et al., 2019) have been reported to cooperate with BRG1 to regulate transcription raising the intriguing question as to how BRG1 selectively interacts with different TETs to regulate transcription. One possible explanation is the availability of different TETs in a given cell type because TET proteins clearly exhibit show distinct expression patterns (Tan and Shi, 2012). For instance, TET1 is the predominant TET isoform detected in HepG2 cells (Chuang et al., 2015). Alternatively, the interaction between BRG1 and different TETs may be fine-tuned by structural components of the SWI/SNF complex (Long et al., 2013; Chandru et al., 2018). Clear delineation of the dynamic interaction between BRG1 and TET proteins, which clearly goes beyond the scope of the present study, is of tremendous help to better appreciation of the underpinnings of transcriptional regulation by the epigenetic machinery.

In summary, our data unveil a novel epigenetic pathway that contributes to galectin-3 trans-activation during liver injury. Further studies using animal models will not only validate the current working model (Figure 5G) but provide further rationale for using this BRG1-TET1 complex as a druggable target for the intervention of liver injuries.

## Data Availability Statement

The raw data supporting the conclusions of this manuscript will be made available by the authors, without undue reservation, to any qualified researcher.

## Ethics Statement

The animal study was reviewed and approved by the Nanjing Medical University Ethics Committee on Humane Treatment of Experimental Animals.

## Author Contributions

YX and MF conceived the project. ZL, FL, MF, CJ, and YX designed the experiments. ZL, FL, QW, CJ, and MF performed the experiments, collected the data, and analyzed the data. CJ, CD, and MF provided funding and supervised the project. YX wrote the manuscript with inputs from all authors.

## Conflict of Interest

The authors declare that the research was conducted in the absence of any commercial or financial relationships that could be construed as a potential conflict of interest.
