# Supplementary material for: Activation of Galectin-3 (LGALS3) Transcription by Injurious Stimuli in the Liver Is Commonly Mediated by BRG1
Source: Front Cell Dev Biol. 2019 Nov 26;7:310. doi: 10.3389/fcell.2019.00310 (PMC6901944; doi:10.3389/fcell.2019.00310)
Supplement: Supplementary file 1 [file Data_Sheet_1.PDF]

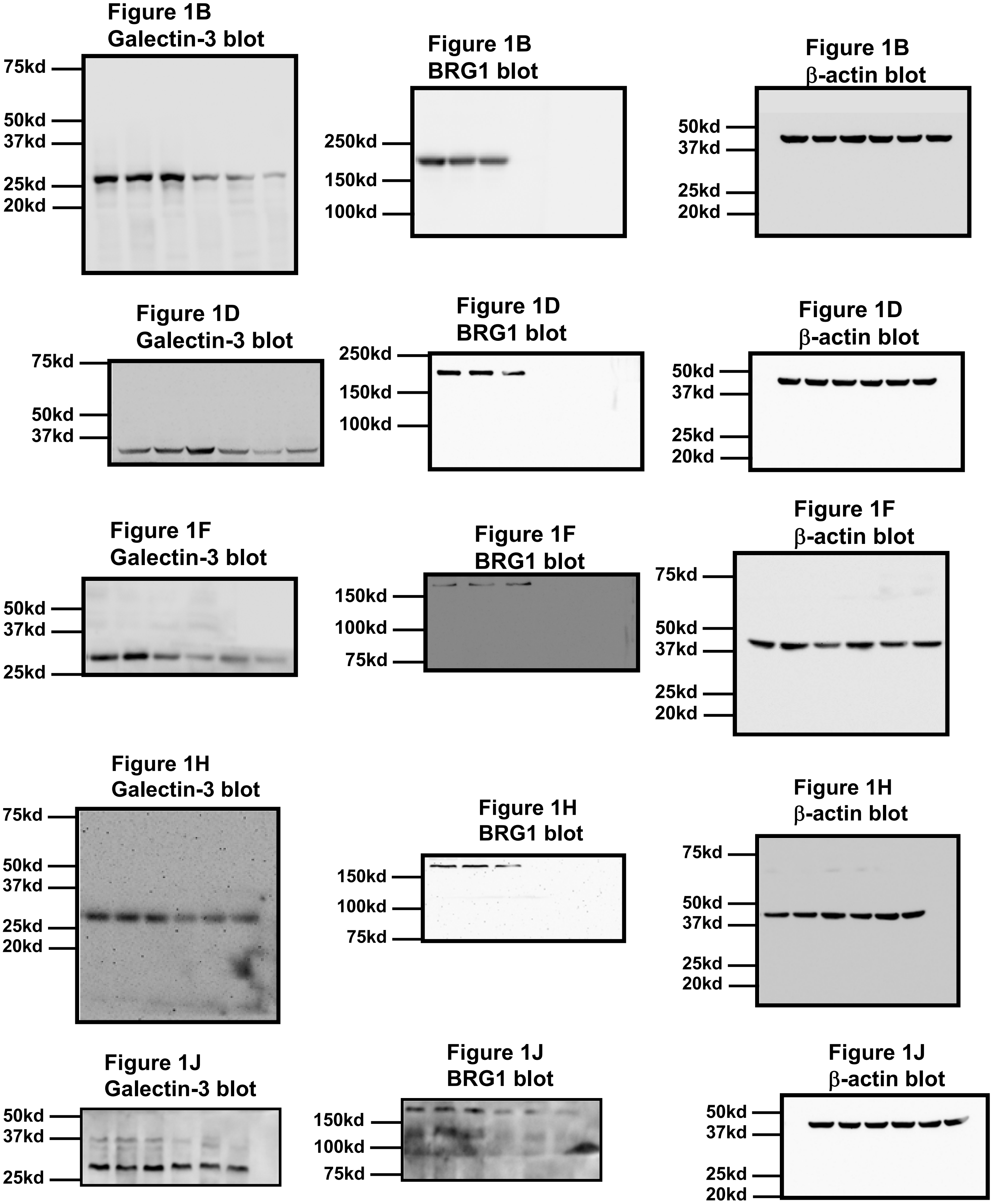

Supplementary Figure 1: Uncropped Western blots for main Figure 1.

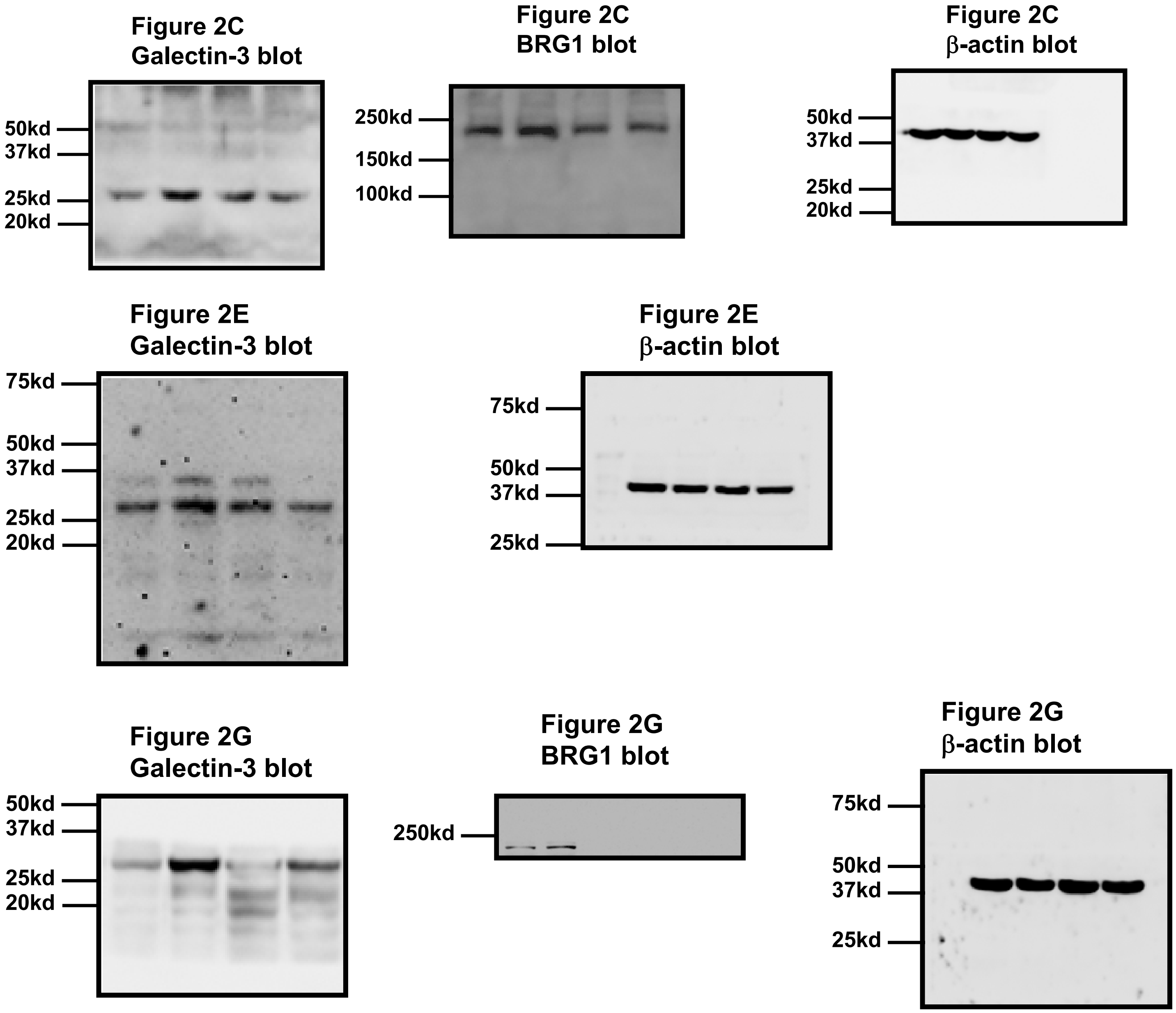

Supplementary Figure 2: Uncropped Western blots for main Figure 2.

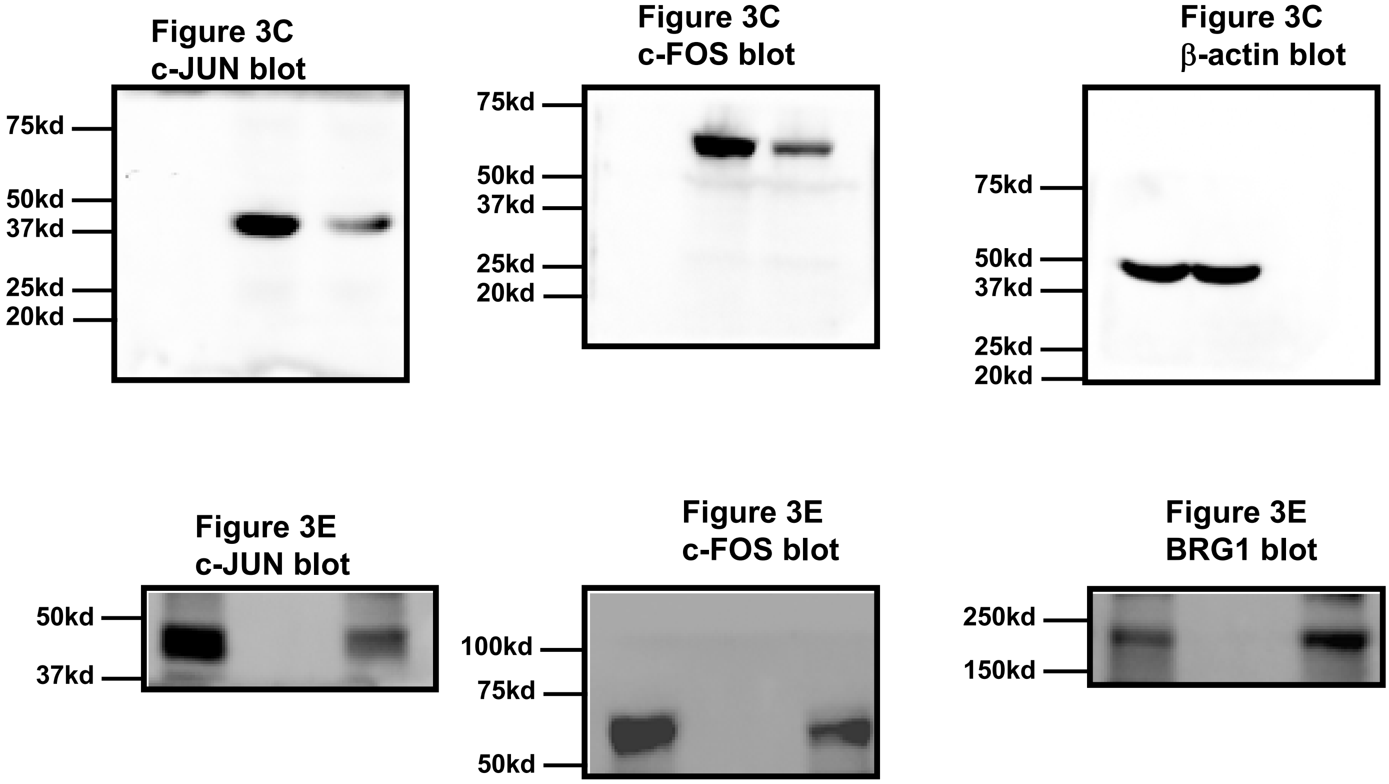

Supplementary Figure 3: Uncropped Western blots for main Figure 3.

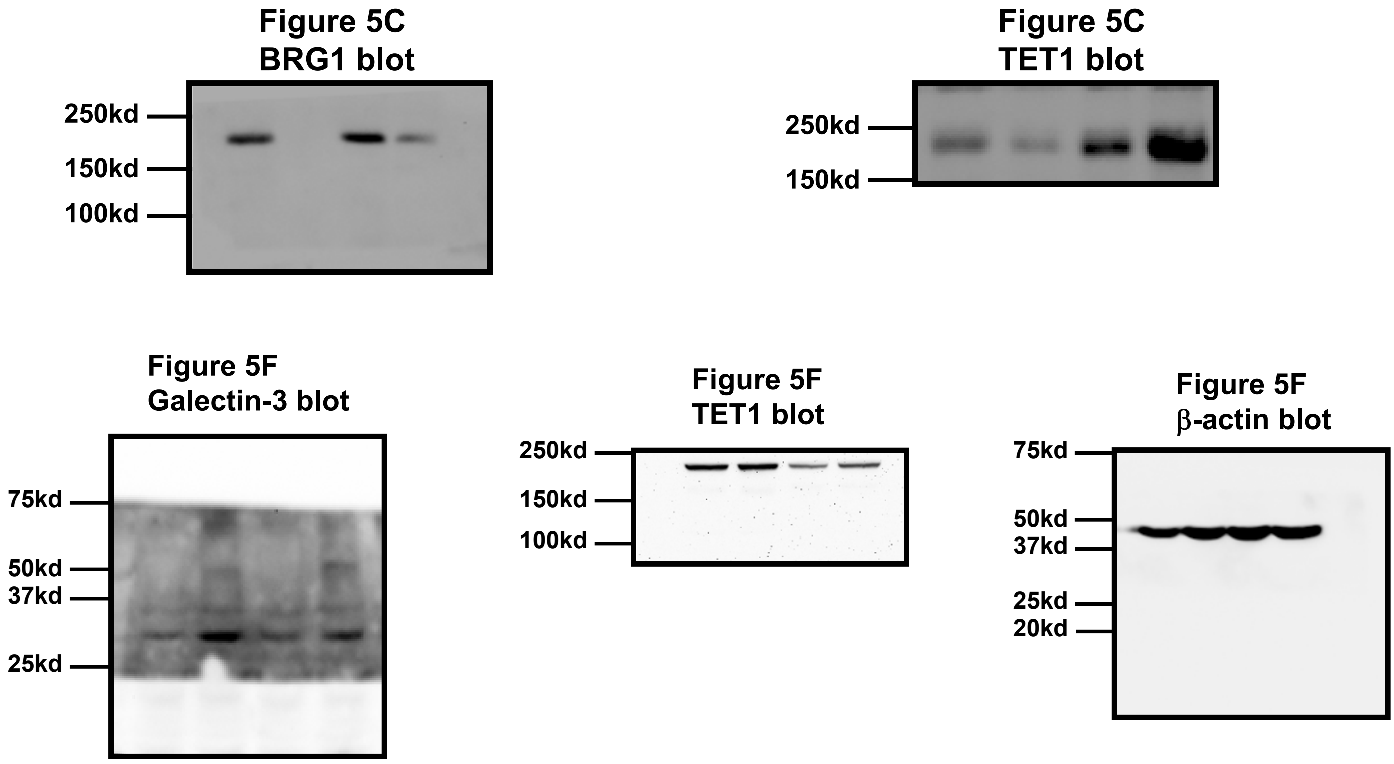

Supplementary Figure 4: Uncropped Western blots for main Figure 5.
